# Unique case of lymphocytic hypophysitis with normal pituitary hormone serology mimicking a non-functioning pituitary adenoma

**DOI:** 10.1186/s12902-024-01546-z

**Published:** 2024-02-08

**Authors:** Kyle Shen, Catherine Cadang, Daniel Phillips, Varsha Babu

**Affiliations:** 1 UCSF, School of Medicine, San Francisco, California USA; 2 UCSF Fresno, Department of Internal Medicine, Fresno, California USA; 3 Pathology Associates, Clovis, CA USA; 4 UCSF Fresno, Division of Endocrinology, Department of Internal Medicine, Fresno, California USA

**Keywords:** Lymphocytic hypophysitis, Autoimmune hypophysitis, Pituitary adenoma

## Abstract

**Background:**

Lymphocytic hypophysitis is a rare autoimmune condition that usually presents during pregnancy and causes inflammation of the pituitary gland. Although the pathophysiology is not well understood, it often presents with headaches, visual disturbances, and symptoms of hypopituitarism. However, not all cases may present with hypopituitarism which can make this rare disease with an incidence of ~ 1 in 9 million much more difficult to diagnose.

**Case Presentation:**

We present a 35-year-old G4P4 woman with progressive vision loss and intermittent frontal headaches during her first trimester through 2 months postpartum. She presented with no symptoms of hypopituitarism and her hormone panel only showed elevated prolactin, possibly due to her breastfeeding. She was treated with a right pterional craniotomy with decompression of both optic nerves, partial resection of the suprasellar mass, and glucocorticoid therapy for headaches and visual disturbances.

**Conclusion:**

This case is notable for a presentation of lymphocytic hypophysitis without symptoms of hypopituitarism. This is important for outpatient providers to be aware of, especially those that care for pregnant patients so that unfavorable outcomes can be avoided.

## Background

Hypophysitis, a rare condition with an incidence of ~ 1 in 9 million, causes inflammation of the pituitary gland. This condition can be caused by primary hypophysitis through lymphocytic, IgG4-related, granulomatous, or neoplastic hypophysitis [1, 2]. It may also be caused by secondary hypophysitis through systemic diseases such as autoimmune conditions, infection, or immunotherapy.

Lymphocytic hypophysitis, also referred to as autoimmune hypophysitis, is the most common cause of primary hypophysitis and presents during or after pregnancy with headaches and visual disturbances due to the pituitary’s mass effect on the optic chiasm. Inflammation of the pituitary gland alone can lead to hypopituitarism. Although the underlying pathogenesis is unclear, autoimmunity plays a large role and histology will show infiltration of lymphocytes in the pituitary biopsy [3, 4]. Lymphocytic hypophysitis often coincides with other autoimmune disorders such as thyroiditis and autoimmune adrenalitis. Based on series of case reports, the estimated prevalence of these autoimmune disorders coinciding are 15–25% and 5–7% respectively [5].

Due to the rarity of this condition, it can be difficult to diagnose especially when symptoms of hypopituitarism are not present. This report presents the case of a patient who presented 7 weeks after delivery with symptoms limited to headaches and visual disturbances due to lymphocytic hypophysitis.

## Case presentation

**Fig. 1:**
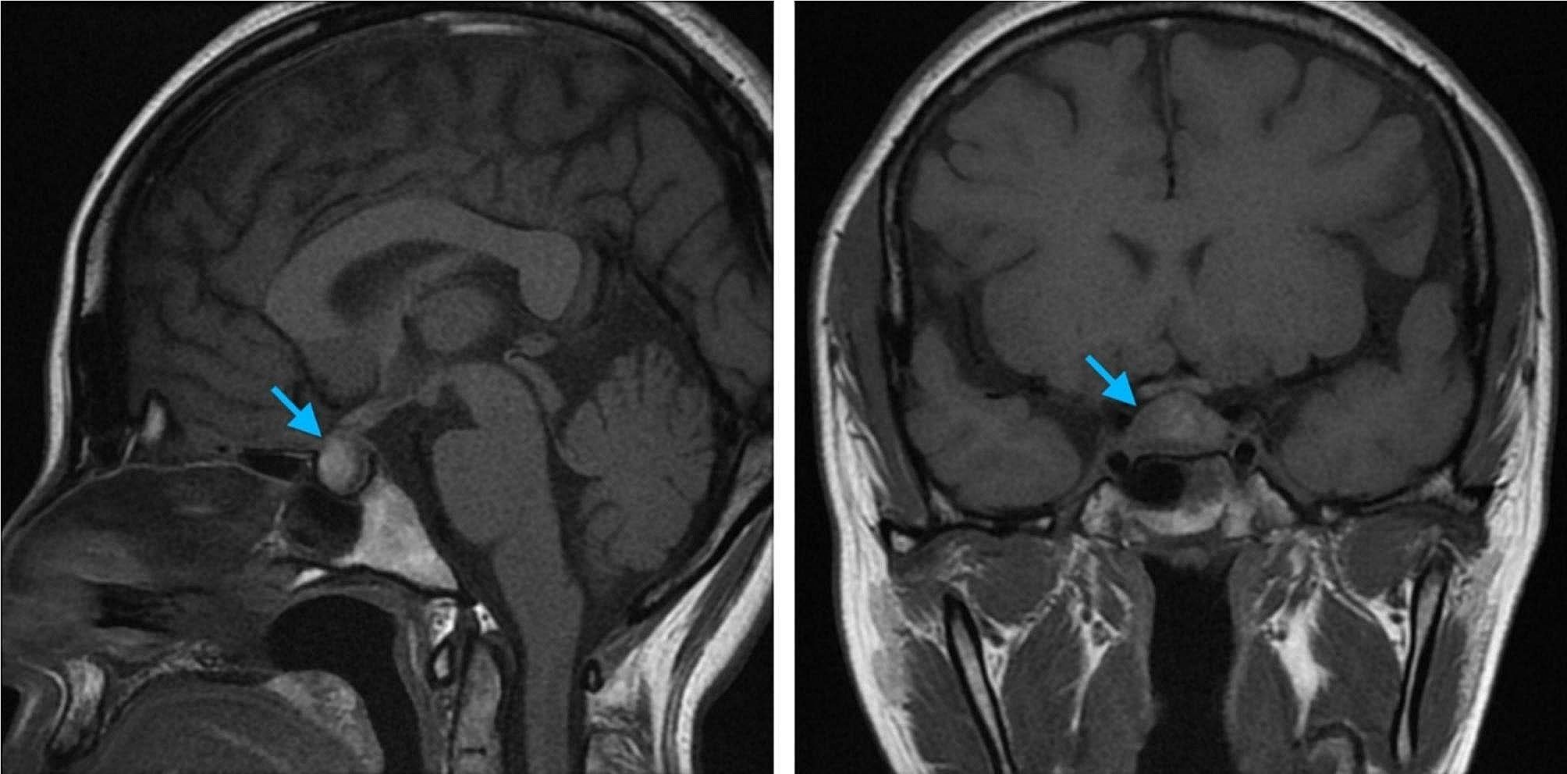
Blue arrow points to the inflamed pituitary gland, pre-op MRI. (**A**) Sagittal view of head MRI T1 without gadolinium contrast. Loss of posterior pituitary signal is present. (**B**) Coronal view of head MRI T1 without gadolinium contrast

**Fig. 2:**
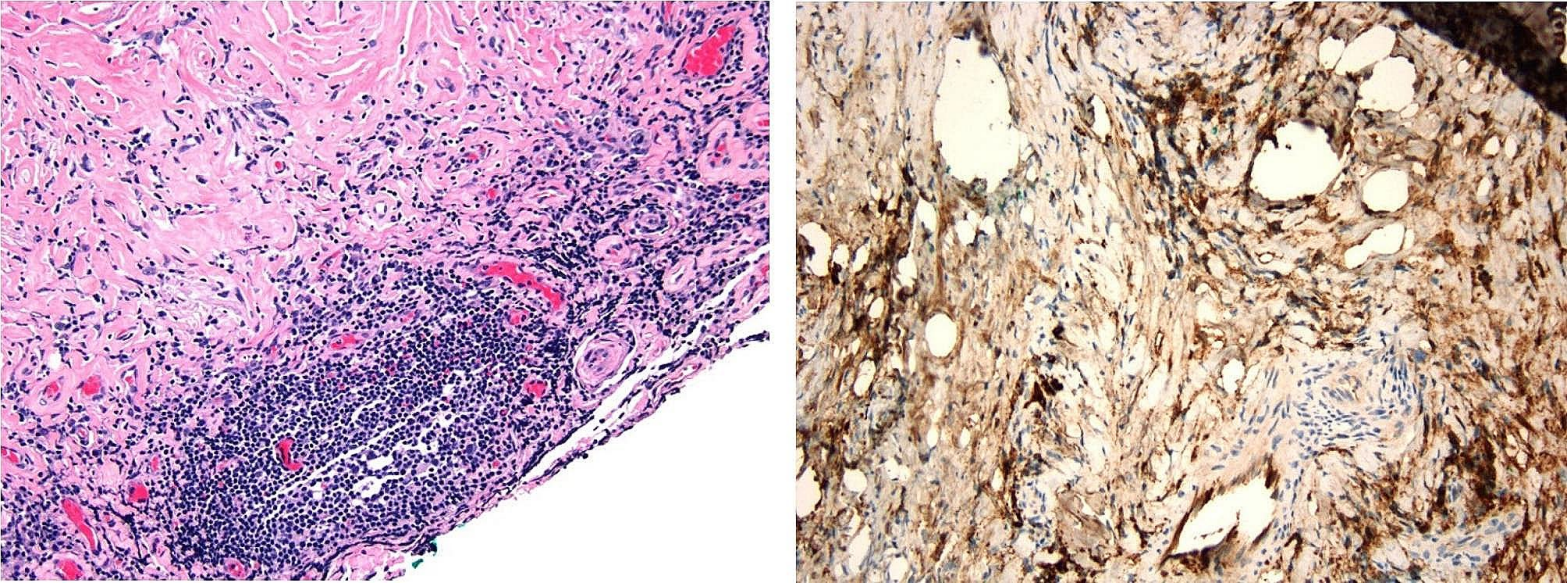
Histopathology images of pituitary biopsy. (**A**) H&E staining at 200x magnification showing dense reactive fibrosis and plasma cells identifiable by the presence of a perinuclear hof. (**B**) IgG4 staining. IgG4 stains often have high background and can be difficult to interpret [6]. This stain was interpreted as negative for IgG4- related disease

This case reviews a 35-year-old G4P4 female with no significant past medical history who initially became symptomatic during her first trimester of pregnancy with progressive vision loss in the right eye with associated intermittent frontal headaches, which persisted throughout pregnancy. After delivery, the patient had scheduled an ophthalmology appointment, but her visual deficits continued to worsen. Seven weeks postpartum, she then presented to our emergency department and was hospitalized for complete vision loss in the right eye and worsening vision in the left eye. Notably, the patient did not have any symptoms of adrenal insufficiency (no hyperpigmentation, salt cravings, nausea, or weight loss) or symptoms of diabetes insipidus (no polyuria or polydipsia). Physical examination revealed pupils equally round, reactive to light, and accommodation, extraocular movements intact, complete vision loss in the right eye with mild proptosis, and only having light perception in the left eye. The rest of the physical examination was unremarkable. Vital signs were within normal limits except for mildly elevated blood pressure at 130/75. Initial lab results were notably within the normal range, except for elevated prolactin possibly due to the patient breastfeeding (Table 1). MRI of the head with and without contrast revealed enlargement of the pituitary gland exerting mild mass effect beneath the surface of the optic chiasm (Fig. 1). Dedicated pituitary MRI revealed a suprasellar hypoenhancing 12.7 mm x 21 mm x 8 mm mass suspicious for pituitary macroadenoma and effacement of the right cavernous sinus. The patient had a normal pituitary hormonal axis including morning cortisol levels on initial evaluation when she was admitted to the hospital. The patient underwent image guided frameless stereotactic right pterional craniotomy with biopsy, decompression of both optic nerves by bone/dura opening of optic canals, and partial resection of the suprasellar mass. Pathology of the biopsy showed dense reactive fibrosis with associated mixed lymphoplasmacytic inflammation and negative IgG4 immunohistochemical staining (Fig. 2). Flow cytometry showed mostly CD45 negative events/debris (85%) due to reduced sample viability, CD45 + lymphocytes (14%) and CD11b + granulocytes (1%).Of the lymphocyte population, CD3 + T-cells had a CD4:CD8 ratio of 1.3 while CD20 + B cells were predominately polytypic/polyclonal. Over the next three days, the patient’s vision improved in both eyes but was blurry, and the right eye was worse than the left eye.

Endocrinology was consulted at the end of the patient’s hospitalization after the patient’s surgery and biopsy result suggesting lymphocytic hypophysitis. We initiated high dose pulse steroid therapy for continued symptoms of vision loss. We also checked T3 and T4 levels which were both within normal limits, since they had not been measured before the surgery. Upon discharge she was initiated on prednisone 50 mg daily for one week and instructed to taper to 40 mg daily for the following week.

**Fig. 3:**
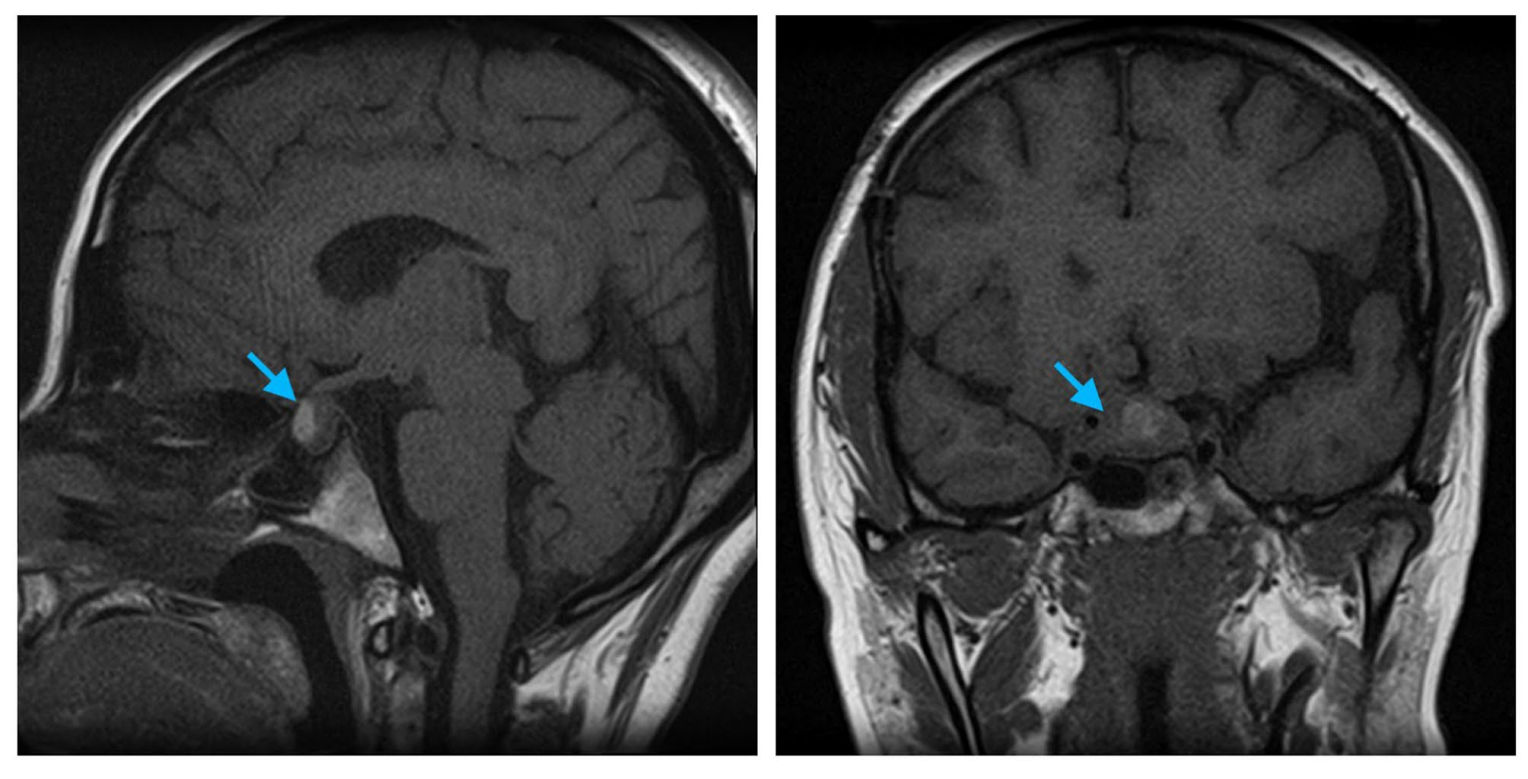
Blue arrow points to the inflamed pituitary gland, 6-month post-op MRI. Compared to pre-op MRI, post-op MRI shows a redemonstration of a sellar mass, similar in size, with mild mass effect on the optic chiasm. (**A**) Sagittal view of head MRI T1 without gadolinium contrast. (**B**) Coronal view of head MRI T1 without gadolinium contrast

At the outpatient endocrinology follow up 2 weeks after discharge, the patient’s only active symptom was persistent blurry vision in the right eye. The patient was tapered off of prednisone over 2 months, and at 6-month follow-up the patient reported her vision was stable with no other symptoms that would indicate Addison’s disease or diabetes insipidus (e.g. no nausea, hypotension, polyuria, or polydipsia). The patient’s 8 AM cortisol and sodium were also both within normal limits (Table 1). At 6-month follow-up with ophthalmology, the patient’s Snellen exam showed L eye was 20/500 and R eye was able to count fingers. The 6-month post-op pituitary MRI revealed redemonstration of a sellar mass, similar in size compared to the pre-op MRI, indicating a surgical outcome of incomplete resection. (Fig. 3).

The expected outcome of the treatment plan is to continue monitoring vision and to monitor for any subsequent endocrine disorders caused by the surgical resection or recurrence of lymphocytic hypophysitis.

**Table 1:** Patient laboratory values before and after surgery

Table of Patient’s Laboratory Values				
Hormones	Range	Value (before surgery)	Value (3 days post-op)	Value (> 4 months post-op)
Sodium	135–145 mmol/L	140	138	142
Potassium	3.5–5.3 mmol/L	3.7	3.7	3.9
Serum Osmolality	282–300 mosm/kg	288	286	283
TSH	0.450–5.330 u[IU]/mL	1.19	0.189↓	1.08
Free T3	2.3–4.2 pg/mL		3	
Free T4	0.8–1.8 ng/dL			0.9
Total Thyroxine	4.5–10.9 ug/dL		8.5	
ACTH	6–50 pg/mL	18		
Cortisol	3.09–22.40 µg/dL	7.78		13.3
GH	≤ 7.1 ng/mL	0.2		
IGF-1	53–331 ng/mL	70		
LH	Follicular Phase: 1.9–12.5 mIU/mL Mid-Cycle Peak: 8.7–76.3 mIU/mL Luteal Phase: 0.5–16.9 mIU/mL Postmenopausal: 10.0–54.7 mIU/mL	0.6		
FSH	Follicular Phase: 2.5–10.2 mIU/mL Mid-Cycle: 3.1–17.7 mIU/mL Luteal Phase: 1.5–9.1 mIU/mL Postmenopausal: 23.0–116.3 mIU/mL	1.9		
Prolactin	Non-pregnant: 3.0–30.0 ng/mL Pregnant: 10.0–209.0 ng/mL Postmenopausal: 2.0–20.0 ng/mL	90.2↑		
Glucose	70–99 mg/dL	98		
ANA screen	negative	negative		

## Discussion

Lymphocytic hypophysitis is a rare autoimmune inflammatory condition affecting the pituitary gland that usually presents with headache, visual disturbances, and hypopituitarism. This case reports a rarer presentation in which the patient’s serology revealed normal pituitary function despite a significant mass effect of the inflamed pituitary gland leading to headaches and visual disturbances. Searching in the PubMed database using the Medical Subject Headings (MeSH) for lymphocytic hypophysitis and filtering for case reports yielded 81 results. Only one case report was found with a similar presentation, where a 28-year-old nonpregnant female with lymphocytic hypophysitis presented with headache, visual disturbances, but normal pituitary function [7]. From an analysis of 492 cases of lymphocytic hypophysitis, 58% had headaches and/or visual disturbances, 44% had signs of hypopituitarism (most commonly decreased ACTH), 31% had polyuria/polydipsia, and 18% had hyperprolactinemia [8]. Imaging findings suggestive of hypophysitis are an enlarged triangular/dumbbell-shaped gland, symmetrical suprasellar gland extension, and homogeneous contrast enhancement [4]. Because lymphocytic hypophysitis presents similarly on imaging to pituitary adenoma, a case-control study of 402 patients created a scoring system to differentiate lymphocytic hypophysitis and non-secreting pituitary adenoma [9]. A score ≤ 0 suggests lymphocytic hypophysitis, and our patient scored a -4. While the pathogenesis of lymphocytic hypophysitis is unknown, it is hypothesized that pituitary autoantigens cause this autoimmune process to occur [3]. However, many of these suspected pituitary autoantigens (such as chorionic somatomammotropin) do not have adequate accuracy for diagnostic testing.

Currently, there are no large randomized clinical trials for the treatment of lymphocytic hypophysitis [10]. When severe visual disturbances are present, treatment often requires total or partial resection of the pituitary through a craniotomy or a transsphenoidal surgery [5, 11, 12]. Glucocorticoid therapy may also be used before or after surgery to decrease swelling and prevent recurrence of hypophysitis [3]. If the patient has no visual disturbances or compression of nearby structures, glucocorticoids may be used alone. Based on a meta-analysis of 17 studies, 36% of lymphocytic hypophysitis patients were treated with steroids while 34% were treated surgically [13]. Other alternative treatments are immunosuppressive medications and radiosurgery with radiotherapy [14]. Prognosis after treatment is variable, although most patients will need to take long-term hormone replacement due to the resection from surgery or recurrence of hypophysitis [6]. A main cause of mortality is adrenal insufficiency, which should be closely monitored for.

The novel findings of this report is that although this patient exhibited visual disturbances and headache, she had no symptoms or unaccountable lab findings of an abnormal pituitary hormonal axis. The patient’s elevated prolactin is within the expected range for a postpartum breastfeeding individual, and the borderline low FSH and LH are within normal limits for the luteal phase which the patient was at the time (the patient reported the start of menses at the end of her hospitalization) [15]. Although the patient would have benefitted from further dynamic hormonal axis testing, post-op labs > 4 months are unremarkable, with a morning cortisol > 13 indicating a normal corticotroph axis [16]. Due to the lack of abnormal hormonal findings, the patient was suspected of having a pituitary macroadenoma which led to surgery as the initial treatment. Only until after the pathology results returned was lymphocytic hypophysitis suspected and further treated with steroids.

## Conclusion

In conclusion, it should be noted that although lymphocytic hypophysitis usually presents with headache, visual changes, and hypopituitarism, it may present with these symptoms but no evidence of hypopituitarism. It is particularly important that providers in the outpatient setting, both primary care providers and OBGYNs, keep this within their differential and consider performing a MRI which is not contraindicated in pregnancy. Furthermore, early intervention with glucocorticoids can prevent progression of visual disturbances and the need for surgery.

## Data Availability

Not applicable.
